# Genome-wide analysis of DNA methylation profile identifies differentially methylated loci associated with human intervertebral disc degeneration

**DOI:** 10.1371/journal.pone.0222188

**Published:** 2019-09-12

**Authors:** Akihiro Ikuno, Koji Akeda, Shin-ichiro Takebayashi, Motomu Shimaoka, Katsuzumi Okumura, Akihiro Sudo

**Affiliations:** 1 Laboratory of Molecular & Cellular Biology, Graduate School of Bioresources, Mie University, Tsu, Japan; 2 Department of Orthopaedic Surgery, Mie University Graduate School of Medicine, Tsu, Japan; 3 Department of Molecular Pathobiology3, Mie University Graduate School of Medicine, Tsu, Japan; University of Iowa Hospitals and Clinics, UNITED STATES

## Abstract

**Background:**

Environmental and endogenous factors under genetic predisposition are considered to initiate the human intervertebral disc (IVD) degeneration. DNA methylation is an essential mechanism to ensure cell-specific gene expression for normal development and tissue stability. Aberrant epigenetic alterations play a pivotal role in several diseases, including osteoarthritis. However, epigenetic alternations, including DNA methylation, in IVD degeneration have not been evaluated. The purpose of this study was to comprehensively compare the genome-wide DNA methylation profiles of human IVD tissues, specifically nucleus pulpous (NP) tissues, with early and advanced stages of disc degeneration.

**Methods:**

Human NP tissues were used in this study. The samples were divided into two groups: early stage degeneration (n = 8, Pfirrmann’s MRI grade: I-III) and advanced stage degeneration (n = 8, grade: IV). Genomic DNA was processed for genome-wide DNA methylation profiling using the Infinium MethylationEPIC BeadChip array. Extraction of raw methylation data, clustering and scatter plot of each group values of each sample were performed using a methylation module in GenomeStudio software. The identification of differentially methylated loci (DMLs) and the Gene Ontology (GO) analysis were performed using R software with the ChAMP package.

**Results:**

Unsupervised hierarchical clustering revealed that early and advanced stage degenerated IVD samples segregated into two main clusters by their DNA methylome. A total of 220 DMLs were identified between early and advanced disc degeneration stages. Among these, four loci were hypomethylated and 216 loci were hypermethylated in the advanced disc degeneration stage. The GO enrichment analysis of genes containing DMLs identified two significant GO terms for biological processes, hemophilic cell adhesion and cell-cell adhesion.

**Conclusions:**

We conducted a genome-wide DNA methylation profile comparative study and observed significant differences in DNA methylation profiles between early and advanced stages of human IVD degeneration. These results implicate DNA methylation in the process of human IVD degeneration.

## Introduction

Low back pain (LBP) is a debilitating disorder that is significantly associated with personal, social, and economic burdens. Recent reports in the Global Burden of Disease (GBD) Study 2015 showed that 7.3% of the global population (540 million people) had activity-limiting LBP on the global point prevalence survey[1].

Epidemiological and clinical studies have recently provided evidence that LBP has a significant association with lumbar intervertebral disc (IVD) degeneration[2–6].

The vertebral column complex consists of ventrally located vertebral bodies and intervening intervertebral discs (IVDs). The IVD is composed of a central gelatinous nucleus pulposus (NP) and a surrounding fibrous anulus fibrosus (AF).

Intervertebral disc degeneration is suggested to be defined as ‘the structural and functional failure of the disc as a result of aberrant, pathological cellular and extracellular matrix (ECM) changes'[7]. The pathophysiology of IVD degeneration is not entirely understood; however, environmental and endogenous factors under genetic predisposition are considered to initiate the degenerative changes of human IVDs (see review in[8]). Intervertebral disc degeneration is generally believed to be a consequence of increased catabolism of the ECM[8, 9]. Biochemically, IVD degeneration, especially NP degeneration, is well characterized by a change in extracellular matrix molecules (loss of proteoglycan and water content in the NP), resulting in an alteration of the biomechanical properties of IVD tissues. These degenerative changes are considered to induce the disruption of IVD tissues, leading to the degenerative disc diseases that are associated with low back pain[9].

A substantial number of mechanisms are known that regulate gene expression and cell fate persistence, commonly referred to as epigenetics[10]. The most extensively studied epigenetic modulation is DNA methylation[11].

DNA methylation induces changes in gene expression without changing the DNA sequence by adding methyl groups to a cytosine in a CpG-containing nucleotide to form 5-methylcytosine[12]. When methylation is located in gene promoter and enhancer regions, DNA methylation typically acts to silence genes, whereas methylation located in gene body regions usually induce enhanced gene expression[13]. DNA methylation is an essential mechanism to ensure cell-type-specific gene expression for normal development, while aberrant epigenetic alterations have been considered to play a pivotal role in several different diseases, such as cancer and neurodegenerative diseases[14, 15]. Therefore, research in epigenetics, including DNA methylation, can elucidate the key pathological process of several diseases; hence, leading to the identification of a new molecular target for therapeutic intervention.

Osteoarthritis (OA) is a chronic musculoskeletal disease characterized by degradation of articular cartilage; similar biochemical changes have also been found in the pathogenesis of IVD degeneration. The involvement of DNA methylation in the pathogenesis of OA has been increasingly evident, reflected by the growing body of reports on the subject. Cross-Sectional studies of DNA methylation on candidate genes have identified alternations in the methylation status of genes involved in OA pathogenesis[16–23]. More recently, genome-wide DNA methylation studies have shown that there is a distinct methylation profile in OA cartilage compared with healthy cartilage in the hip and knee joints[24–31]. However, epigenetic alternations, including DNA methylation, in IVD degeneration have not been evaluated.

The purpose of this study was to comprehensively compare the Genome-wide DNA methylation profiles of human NP tissues at early and advanced stages of disc degeneration using an Infinium MethylationEPIC BeadChip array.

## Materials and methods

### Human intervertebral disc samples

Study ethics were approved by the institutional review board of the Mie University Hospital (Tsu, Mie, Japan; IRB reference number: H2018-050). Written or oral informed consent was obtained from all participants.

**Fig 1:**
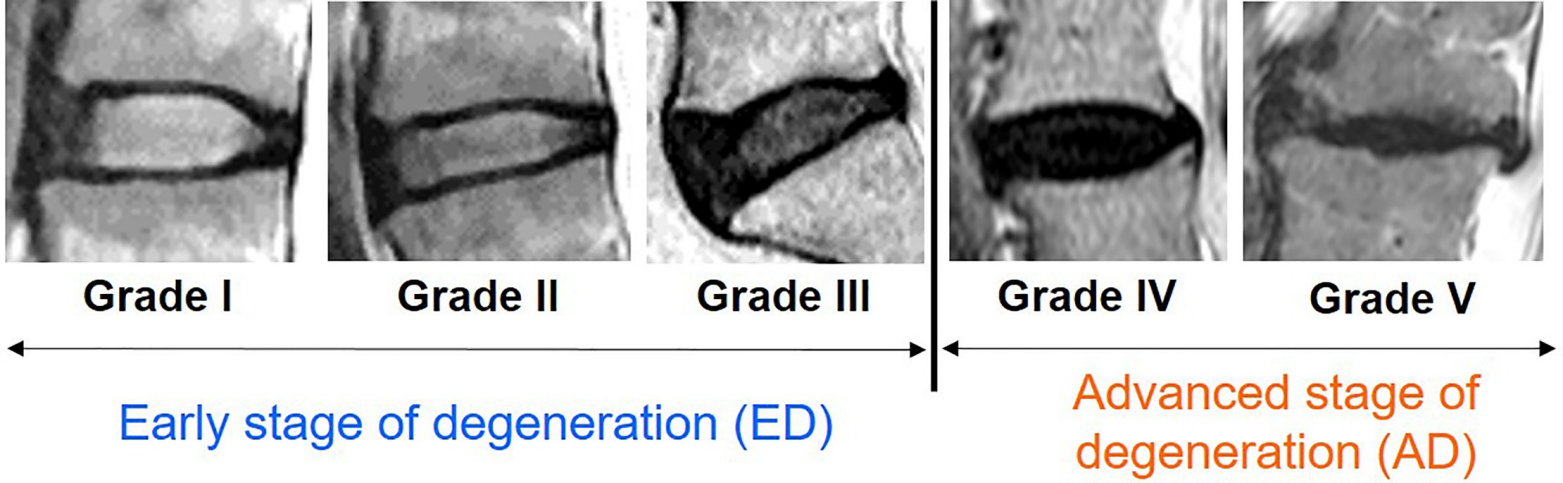
Magnetic resonance imaging (MRI) of human intervertebral disc classified according to the Pfirrmann grading system[22]. Grades I to III were classified as early stage degeneration (ED). Grade IV and V were classified as advanced stage degeneration (AD).

Human IVD tissues obtained from spine surgeries were used in this study (average age: 55.6 [25–83] years-old). The degree of disc degeneration was evaluated by preoperative magnetic resonance imaging (MRI) according to Pfirrmann’s classification[32]: grade I (n = 3); grade II (n = 3); grade III (n = 2); grade IV (n = 8) (Fig 1). Human IVD tissues were divided into two groups: early stage degeneration (Pfirrmann grades I-III, Fig 1) and advanced stage degeneration (Pfirrmann grades IV–V, Fig 1) (Table 1). NP tissues grossly separated from human IVD samples were stored at -80°C until used.

**Table 1:** Patient characteristics.

ID	Age (years)	MRI grade	Grander	Diagnosis
**ED01**	66	III	Male	Degenerative disc disease
**ED02**	38	II	Male	Spinal trauma
**ED03**	39	I	Male	Spinal trauma
**ED04**	39	I	Male	Spinal trauma
**ED05**	25	I	Male	Spinal trauma
**ED06**	63	II	Female	Spinal trauma
**ED07**	34	III	Male	Spinal trauma
**AD01**	52	IV	Female	LSS
**AD02**	71	IV	Female	LSS
**AD03**	83	IV	Female	LSS
**AD04**	64	IV	Female	LDS
**AD05**	67	IV	Female	LDSc
**AD06**	56	IV	Female	LDS
**AD07**	74	IV	Female	LDS
**AD08**	56	IV	Female	LDS

ED: Early stage disc degeneration, AD: Advanced stage disc degeneration, LSS: Lumbar spinal stenosis, LDS: Lumbar degenerative spondylolisthesis, LDSc: Lumbar degenerative scoliosis

### DNA isolation and bisulfate treatment

Frozen NP tissue samples (200–250 mg wet weight) were pulverized in the presence of liquid nitrogen using a cryopress (Microtech Nichion, Chiba, Japan). DNA was isolated using the Wizard® Genomic DNA Purification Kit (Promega, City, WI, USA) according to the manufacturer's instruction. First, protein precipitation solution, 0.5 M EDTA and nuclei lysis solution were added to the sample (20 mg), and the sample was then treated with Proteinase K. Next, DNA was precipitated by adding isopropanol. Finally, the DNA pellet was washed twice in 70% ethanol and resuspended in sterilized ultrapure water. The concentration of DNA was measured by the Qubit® dsDNA HS Assay Kit (Molecular Probes, City, OR, USA). The DNA samples were stored at -20°C. Five hundred ng of genomic DNA was then bisulfite converted using an EZ DNA methylation kit (Zymo Research, Irvine, CA, USA) and eluted in 10 μl of elution buffer (50 ng/μl).

### Genome-wide DNA methylation profiling

DNA methylation profiling was performed on bisulfite-converted genomic DNA in the Center for Molecular Biology and Genetics of Mie University using the Infinium MethylationEPIC BeadChip array, which allowed the interrogation of over 850,000 methylation sites throughout the genome at single-nucleotide resolution (Line #000010, catalog #WG-317-1001, Illumina, San Diego, CA, USA). The arrays were processed following the manufacturer's instructions and scanned in an Illumina iScan (Illumina). Extraction of raw methylation data, scatter plots of each group values and clustering of each sample were performed using the Methylation module (Version 1.90) in GenomeStudio software (V2011.1, Illumina). GenomeStudio provides the methylation data as β values: β = M/(M + U), which were calculated from the fluorescent signal of the methylation probe (M) and unmethylated probe (U). The β values range from 0 (no methylation) to 1 (100% methylation).

A difference in β values between early and advanced IVD degeneration stage groups were tested with the Illumina Custom model. False discovery rate (FDR)—corrected P values and DiffScores were computed. The data of the Infinium MethylationEPIC BeadChip array are available on NCBI NIH Gene Expression Omnibus (https://www.ncbi.nlm.nih.gov/geo/) under accession number # GSE129789. For scatter plot and clustering analyses, raw data were normalized and background was subtracted using a control probe in this array. Probes that had a detection *P*—value greater than 0.01 were removed from the analysis data. Because male and female samples were studied, sex chromosome probes were also removed.

### Data processing and statistical analysis

Processing of the raw methylation data was performed using R (version 3.4.3; https://www.r-project.org/) with the Chip Analysis Methylation Pipeline (ChAMP) package[33]. Raw methylation data were imported by the minfi method[34, 35], and normalized by the SWAN method[36]. By default setting, raw data were filtered for probes with a detection *P* > 0.01, non-CpG site[37], the multi-hit probe list[38] or X and Y chromosomes. As a result, the remaining 741955 probes were utilized for data analysis. To show statistically significant genome-wide differences in the differential methylated loci (DMLs), the adjustment *P*-value (Adjust *P*.Value) moderated with “BH (Benjamini-Hochberg)” correction was calculated using the limma package[39, 40]. DMLs whose Adjust *P*.Value was less than 0.05 were selected. Gene ontology analysis for 220 DMLs was performed using the missMethyl package with the gometh method[41–43].

### Comparison analysis of differentially methylated loci (DMLs) with human knee osteoarthritis

To identify the common gene symbols that differentially methylated between human IVD degeneration and human knee osteoarthritis, 187 gene symbols comprising 220 DMLs identified in this study were compared with 484 gene symbols comprising 653 DMLs identified in knee osteoarthritis (OA) cartilage study[44]. The percentage of common gene symbols against 187 genes and the percentage of the DMLs associated with common gene symbols against 220 DMLs were calculated.

### Statistical analysis

The correlation between methylation β values and age was evaluated using Pearson’s correlation coefficient test. Differences in methylation β values were assessed for statistical significance by two-way analysis of variance (ANOVA) to compare the disc degeneration groups (ED and AD) and gender. All the statistical analyses were performed using IBM Statistical Package for Social Sciences Software (SPSS) Statistics (IBM Japan, Tokyo). The accepted level of significance was p<0.05.

## Results

### DNA methylome in early and advanced stages of human intervertebral disc degeneration

**Fig 2:**
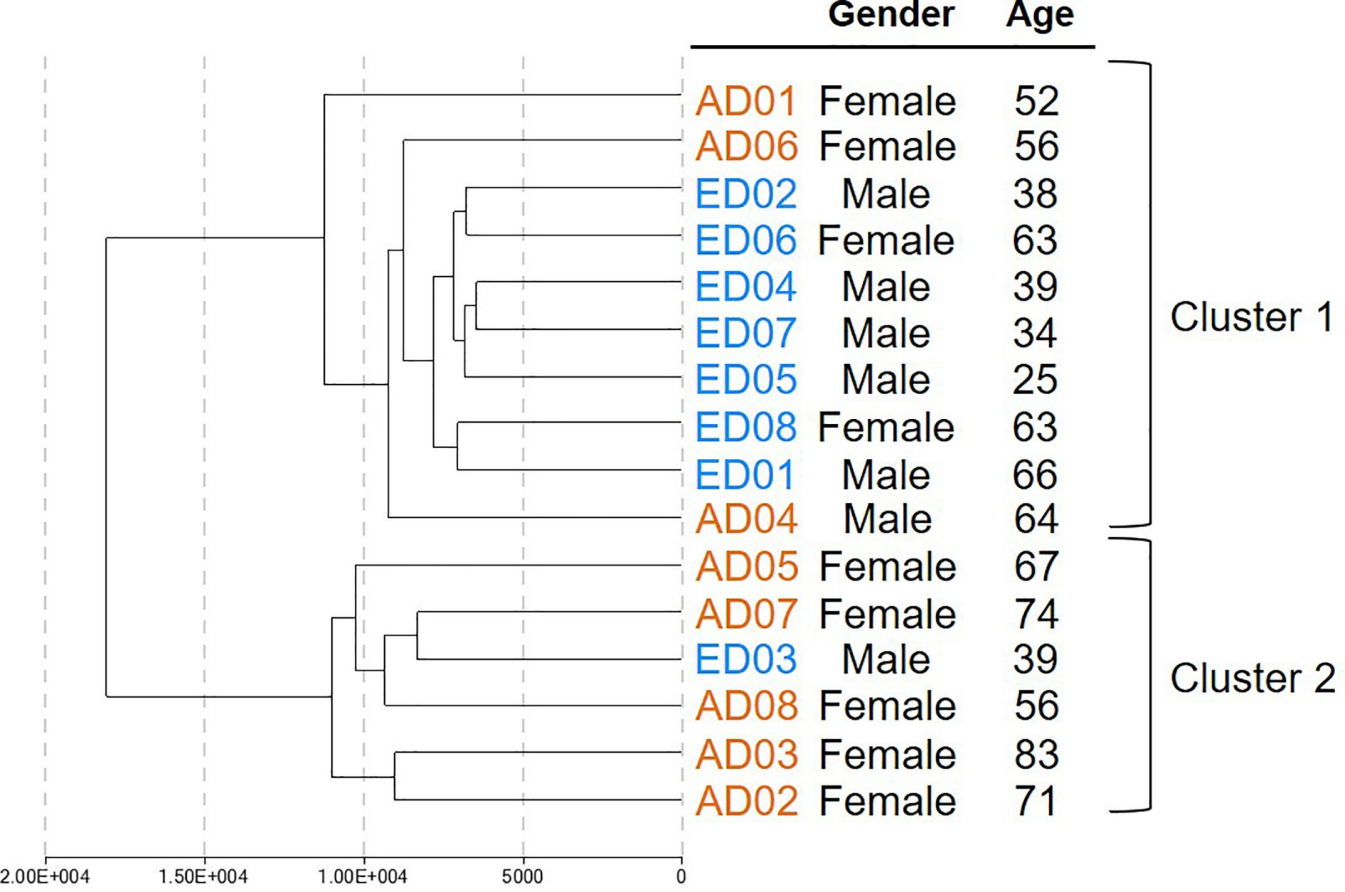
Unsupervised hierarchical clustering of DNA methylation values of human nucleus pulposus (NP) tissues in eight early stage (ED) and eight advanced stage (AD) of disc degeneration.

**Fig 3:**
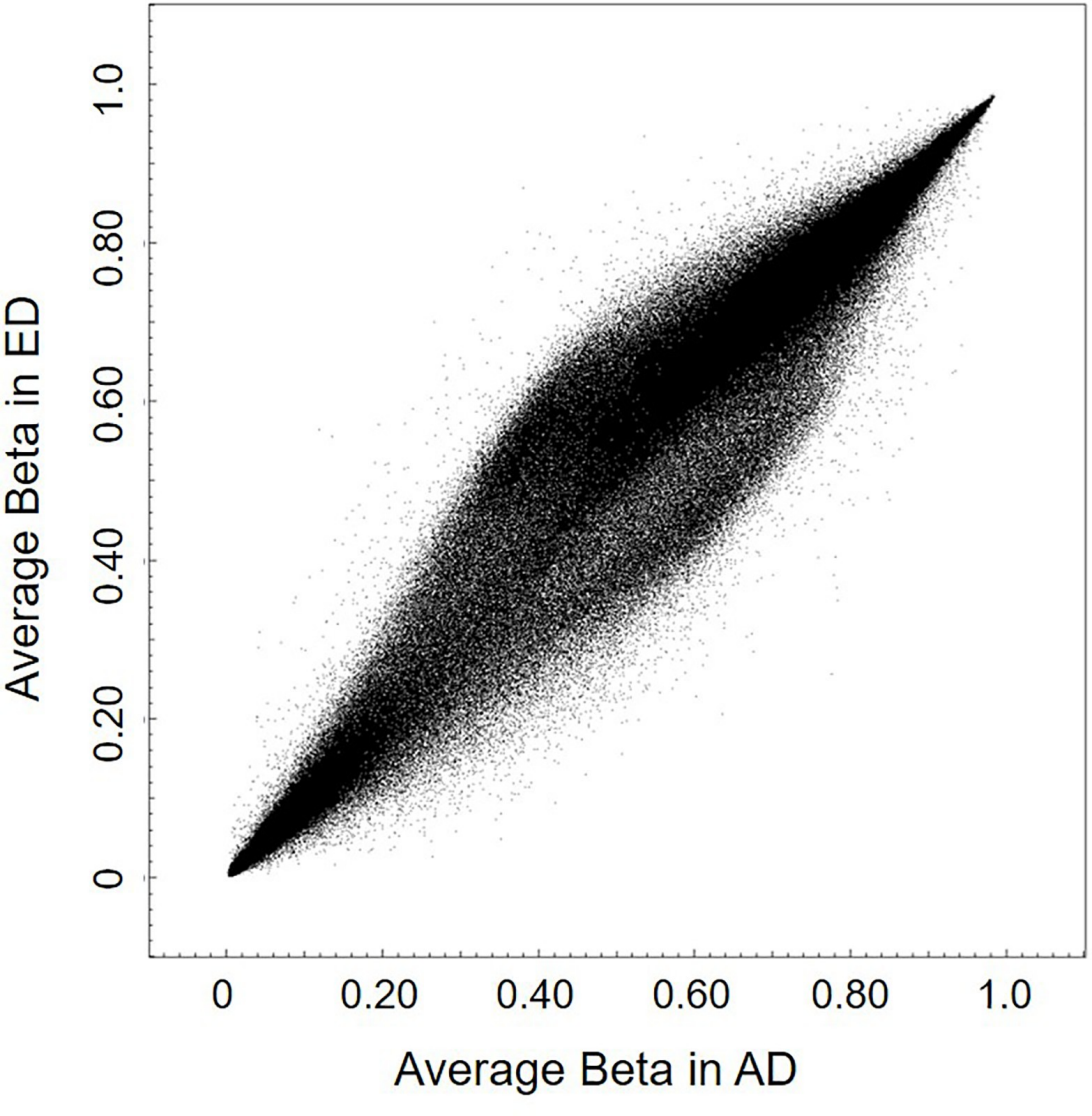
Scatter plot of average DNA methylation level of early stage (ED) and advanced stage (AD) of degeneration.

Unsupervised hierarchical clustering revealed that early and advanced stages of degenerated samples segregated into two main clusters by their DNA methylome (Fig 2). Cluster 1 consists of 7 ED samples and 3 AD samples and cluster 2 consists of 5 AD samples and 1 ED sample. Scatter plot of average methylation β values in all ED and AD samples are presented in Fig 3.

### Identification of differentially methylated loci (DMLs) in early and advanced stages of human disc degeneration

A total of 220 differentially methylated loci (DMLs) were identified in early and advanced IVD degeneration stages, comprising a total of 187 individual genes (for the complete list of DMLs, see supporting information, S1 Table). Among these, four loci were hypomethylated, and 216 sites were hypermethylated in the advanced stage of degenerated IVDs. The gene-associated four hypomethylated DMLs and ten highest hypermethylated DMLs in the advanced IVD degeneration stage are shown in Table 2.

**Table 2:** Analysis of significantly differentially methylated loci (DMLs).

Illumina probe ID	Associated gene	ΔMean Beta	Adjust *P*.Value	Region
**Hypomethylated in AD**				
**cg10846936**	*CARD14*	-0.110885116	0.007781919	Body
**cg09422970**	*CRHR1*	-0.050218804	0.024295548	5'UTR
**cg26175287**	*C14orf139*	-0.044147002	0.031953803	TSS1500
**cg04634182**	*ZBTB47*	-0.083351824	0.048423567	5'UTR
**Hypermethylated in AD**				
**cg00106685**	*GNL3*	0.105363371	0.00029138	1stExon
**cg22777949**	*SNORA52*	0.083679742	0.0005680	TSS1500
**cg24668990**	*XKR5*	0.07712002	0.0005680	Body
**cg11871820**	*MED23*	0.107756592	0.001778816	TSS200
**cg24947371**	*GPR133*	0.165178116	0.002749519	Body
**cg08616760**	*ZNF354A*	0.138590611	0.005013752	TSS200
**cg07740693**	/	0.104746299	0.005013752	IGR
**cg21872822**	*IGF2BP1*	0.085669086	0.005013752	5'UTR
**cg20090957**	*MAPKAPK5*	0.082908954	0.005013752	TSS1500
**cg23725152**	*INAFM1*	0.064246138	0.005013752	Body

AD: advanced stage of intervertebral disc degeneration

**Fig 4:**
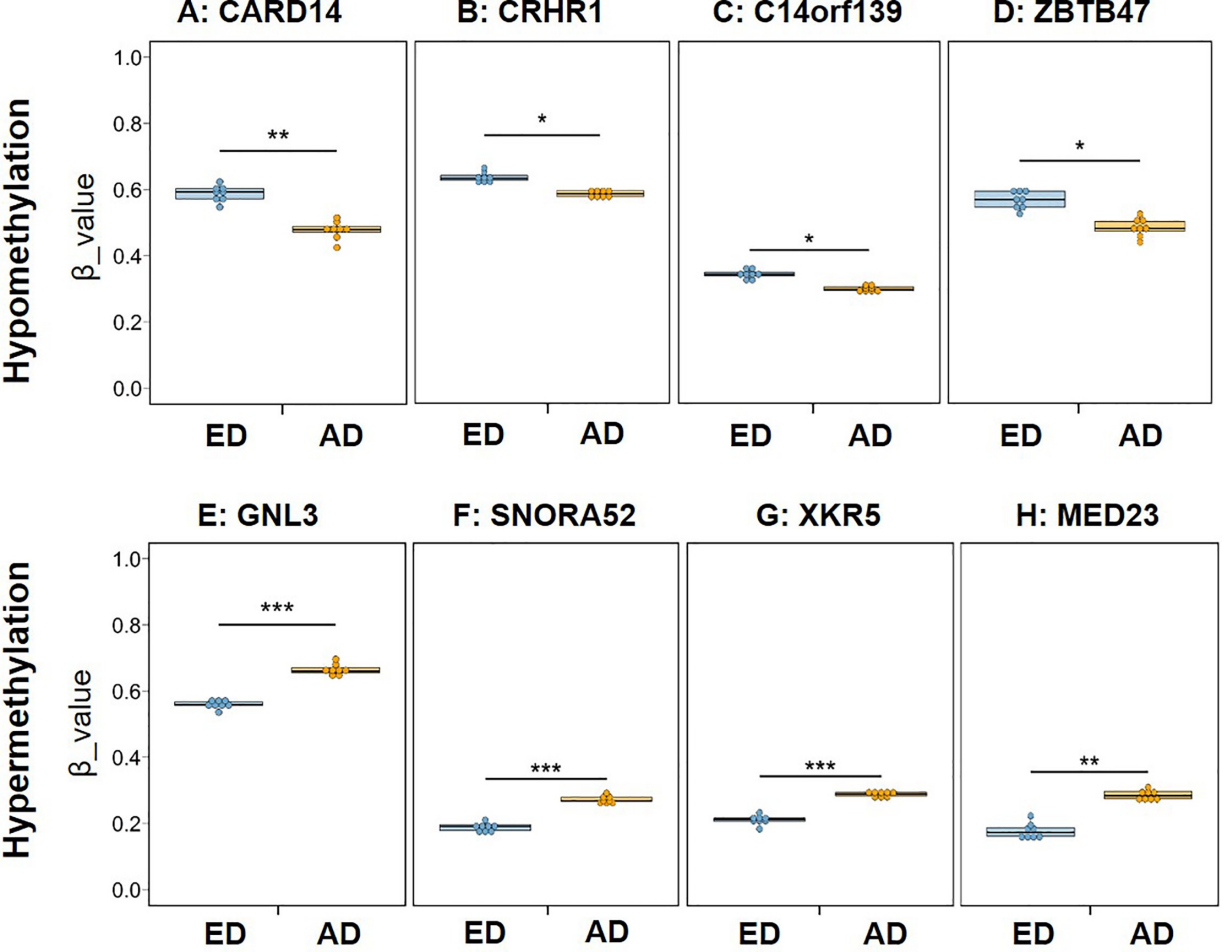
Differences in β value of four highest hypomethylated and hypermethylated loci between early stage (ED) and advanced stage (AD) of degeneration. * = adjust. *P*. Val < 0.05; ** = adjust. *P*. Val < 0.01; *** = adjust. *P*. Val < 0.001. A: *CARD14* (Caspase Recruitment Domain Family Member 14), B: *CRHR1* (Corticotropin Releasing Hormone Receptor 1), C: *C14orf139* (Chromosome 14 Open Reading Frame 139), D: *ZBTB47* (Zinc Finger And BTB Domain Containing 47), E: *GNL3* (G Protein Nucleolar 3), F: *SNORA52* (Small Nucleolar RNA, H/ACA Box 52), G: *XKR5* (X Kell Blood Group Precursor-Related Family, Member 5), H: *MED23* (Mediator Complex Subunit 23).

Examples of methylation β value plots for the four representative hypomethylated and hypermethylated DMLs are shown in Fig 4. In the hypomethylated DMLs, the averaged β value of *CARD14*, *CRHR1*, *C14orf139* and *ZBTB47* in the ED group was significantly higher compared to those in the AD group (Fig 4A–4D). In the hypermethylated DMLs, the averaged β value of *GNL3*, *SNORA52*, *XYR5* and *MED23* in the ED group was significantly lower compared to those in the AD group (Fig 4E–4H).

**Fig 5:**
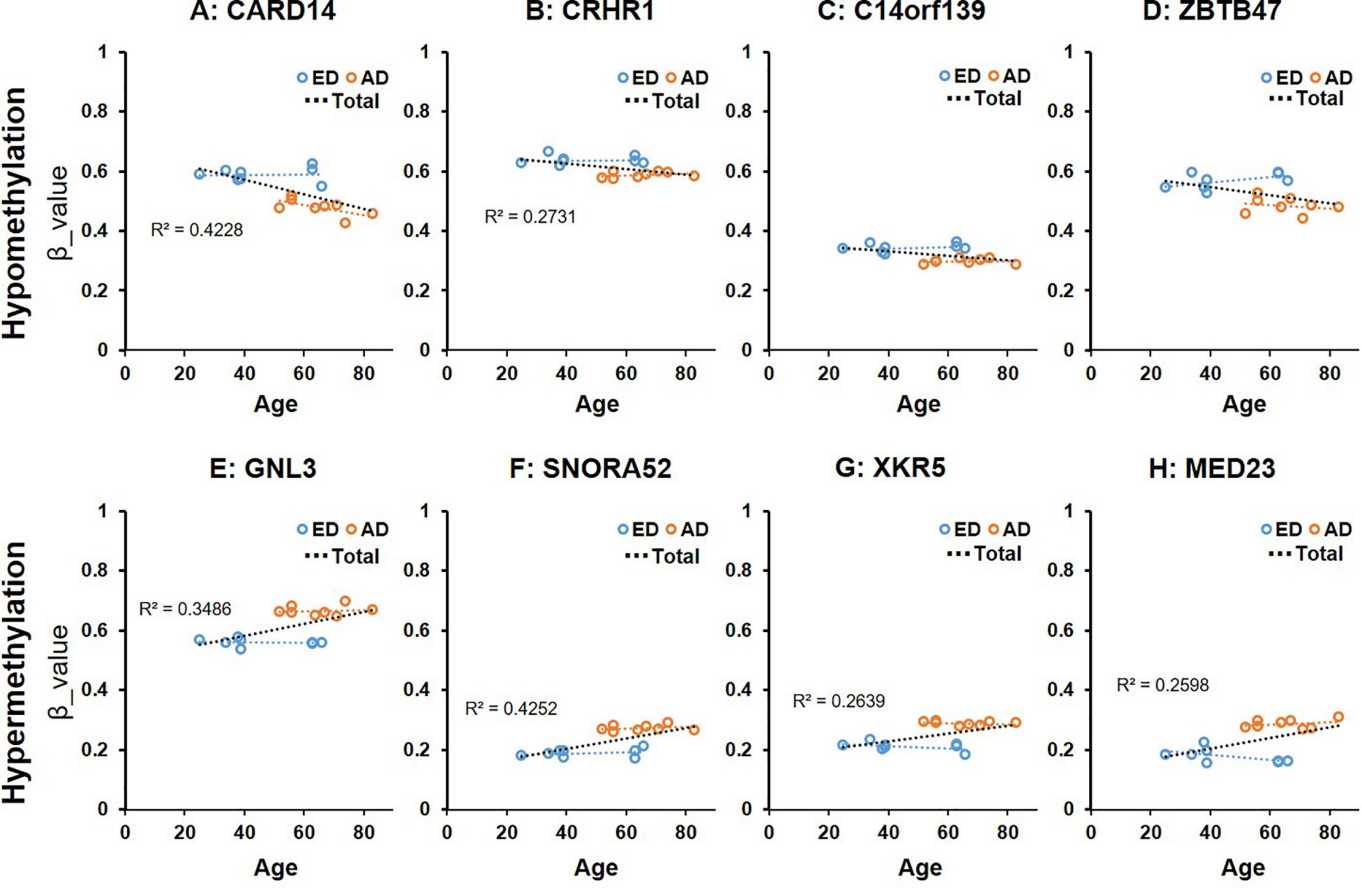
Scatter plots of the β value of four highest hypomethylated and hypermethylated loci between early stage (ED) and advanced stage (AD) of degeneration according to age. Blue dots indicate the β values of the early stage of degenerated (ED) samples, and orange dots indicate β values of the advanced stage of degenerated (AD) samples. R2: correlation coefficient. *P<0.05, **P<0.01. A: CARD14 (Caspase Recruitment Domain Family Member 14), B: CRHR1 (Corticotropin Releasing Hormone Receptor 1), C: C14orf139 (Chromosome 14 Open Reading Frame 139), D: ZBTB47 (Zinc Finger And BTB Domain Containing 47), E: GNL3 (G Protein Nucleolar 3), F: SNORA52 (Small Nucleolar RNA, H/ACA Box 52), G: XKR5 (X Kell Blood Group Precursor-Related Family, Member 5), H: MED23 (Mediator Complex Subunit 23).

In the total eight samples including ED and AD groups, a significant correlation between methylation β-value and age was found in 6 genes (CARD14, CRHR1, GNL3, SNORA52, XKR5, and MED23); however, the remaining two genes (C24orf139 and ZBTB47) showed no significant correlation between methylation β value and age (Fig 5). When the data were analyzed by ED and AD groups, respectively, no significant correlation between methylation β values and age were identified both by ED and AD groups (Fig 5).

For evaluating the involvement of gender on DNA methylation, the β values of these representative eight genes were statistically evaluated. A significant difference in β values by gender was only identified by MED23 (P<0.05, Two-way ANOVA); however, the remaining seven genes showed no significant differences by gender. Furthermore, no significant differences in the interaction effect of disc degeneration (DD) groups and gender were also identified.

### Identification of differentially methylated loci (DMLs) shared between human IVD degeneration and human knee osteoarthritis

When compared to data of 653 DMLs from human knee osteoarthritis cartilage[44], six common genes were identified (Table 3). Among 220 DMLs comprising a total of 187 individual genes found in the advanced stage of disc degeneration IVDs, 2.7% (6/220) DMLs, and 3.2% (6/187) individual genes were shared with those from knee OA cartilage previously reported by Alvarez-Garcia et al.[44].

**Table 3:** Overlap of differentially methylated loci (DMLs) identified in human IVD degeneration and human knee osteoarthritis (OA).

IVD degeneration (Current study)		Knee OA[44]	
Probe	Associated gene	Probe	Associated gene
cg12697442	*YAP1*	cg09612099	*YAP1*
cg03646234	*TMIE*	cg00153306	*TMIE*
cg23039660	*FGFRL1*	cg08521859	*FGFRL1*
		cg16185996	
		cg04145890	
		cg18699025	
		cg07727358	
cg19878849	*NAA25*	cg18700744	*NAA25*
cg14711690	*ITPKB*	cg05306109	*ITPKB*
cg21580428	*PCDHGA4*	cg14566959	*PCDHGA4*

### Gene ontology analysis

The GO enrichment analysis of genes containing DMLs identified two significant GO terms for biological processes associated with cell adhesion; these were hemophilic cell adhesion through plasma membrane adhesion molecules (enrichment 11.2%, *P* = 5.86E-06) and cell-cell adhesion through plasma membrane adhesion molecules (enrichment 7.8%, *P* = 9.86E-05).

## Discussion

This is the first study that compared DNA methylation profiles of the human NP between early and advanced stages of disc degeneration using the comprehensive methylation array, the Illumina Infinium MethylationEPIC array. We identified 220 differentially methylated loci that comprised a total of 187 individual genes, revealing that the early and advanced degenerated human NP tissues exhibit substantially different methylomes. Furthermore, the GO enrichment analysis identified two significant GO terms for biological processes associated with cell adhesion.

As written in the recent review on the biological aging of intervertebral disc[8], the authors described that disc degeneration could be theoretically distinguished from disc aging. Disc aging is considered to occur systemically in all spinal discs of all older individuals. On the other hand, no precise definition of "intervertebral disc degeneration" has been accepted for biomedical research and/or clinical practice. Adams et al.[7] reported that IVD degeneration is considered to be a structural failure with accelerated or advanced changes of the aging disc. Unfortunately, however, the specific biological differences between an aged disc and a degenerated disc have not been clearly defined because both share many similar biological, histological, and radiological changes[7]. Importantly, degenerative disc diseases also should be applied to be a degenerated disc that is also painful and/or associated with neurological symptoms[7].

In the clinical setting, human IVDs with a MRI finding of ‘no clear distinction between the AF and NP', which signifies the loss of signal of the NP on T2-weighted images, the generally accepted image findings of disc degeneration[45, 46], were assigned to Pfirrmann grade IV or V[32] (Fig 1). A previous study demonstrated that the loss of signal intensity in the NP area is significantly associated with the morphological features and biochemical contents of degenerative human IVDs[46]. Furthermore, the expression of catabolic factors, such as proinflammatory cytokines and matrix-degrading enzymes, was upregulated in the degenerated human IVD evaluated by MRI[47–51]. We, therefore, defined an MRI classification of more than grade IV as ‘advanced stage of degeneration.'

Human IVD samples at the early stage of disc degeneration were obtained from anterior fusion surgeries of spinal trauma patients, except for one patient. On the other hand, those at the advanced stage of disc degeneration were obtained from spinal fusion surgeries for patients with degenerative lumbar diseases, such as lumbar spinal stenosis or degenerative spondylolisthesis. It should be kept in mind that the differences in DNA methylation profiles between these two groups, therefore, reflect not only changes in MRI findings, but also the underlying changes with/without lumbar degenerative diseases caused by progressive disc degeneration. Nevertheless, it would not be a practical issue in this study that the human IVD samples isolated from the patients with degenerative lumbar diseases can be regarded as the discs with advanced disc degeneration (AD).

Biochemical characteristics of IVD degeneration, especially those of NP tissues, have been characterized to represent the degradation of the extracellular matrix[9]. The biochemical changes of the major components of the human NP (type II collagen and the proteoglycan aggrecan), and also minor components, including collagen (types III, V, VI, IX-XII and XIV) and small proteoglycans (lumican, biglycan, decorin and fibromodulin), during disc degeneration have been well documented[9, 52]. However, the results of the current study showed no significant changes in DNA methylation profiles in these major and minor matrix components of human NP tissues between early and advanced disc degeneration.

Biologically, IVD cells, including NP cells, regulate the homeostasis of IVD tissues by maintaining a balance between anabolism and catabolism[9]. Therefore, an imbalance between anabolic and catabolic pathways is considered to be responsible for the onset and progression of IVD degeneration.

The progression of IVD degeneration is characterized by increased extracellular matrix degradation by locally produced matrix metalloproteinases (MMPs) and ADAMTSs (a disintegrin and metalloproteinase with thrombospondin motifs), which enzymatically degrade collagens and aggrecan. Importantly, the expression of those matrix-degrading enzymes can be stimulated by locally produced pro-inflammatory cytokines, such as interleukin-1β (IL-1β) and tumor necrosis factor-α (TNF-α)[51, 53–55]. However, the current study showed that MMPs, ADAMTSs, and proinflammatory cytokines were not differentially methylated in the advanced IVD degeneration stage compared to those in the early stage.

Activation of nuclear factor-κB (NF-κB), which plays a central role in inflammation through its ability to induce transcription of proinflammatory genes, including TNF-α, IL-1β, IL-6 and IL-8[56], has been shown to increase disc degeneration by upregulating the expression of matrix-degrading enzymes, such as MMPs and ADAMTSs[57]. Interestingly, we identified three hypermethylated genes in the advanced stage of disc degeneration (*CARD14*[58], *EFHD2* and *RTKN2*[59]) that are involved in the regulation of the NF-κB pathway. Also, hypermethylated genes associated with the MAPK signaling pathway such as *MAPKAPK5*[60, 61] and *PRKCZ*[62] that have the potential to regulate multiple catabolic molecules were identified.

Importantly, the Wnt signaling pathway has also been reported to be associated with extracellular matrix metabolism by regulating pro-inflammatory stimuli. Our results showed that *WNT5A*, one of the Wnt proteins family, was differentially methylated in advanced stage degenerated IVD tissues. Wnt proteins are a major family of signal molecules that regulate cell biological and developmental processes[63]. Wnt proteins and the Wnt signaling pathway have also been implicated in the regulation of inflammatory processes in osteoarthritis and disc degeneration[64–67]. Among Wnt proteins, Wnt-5a, a representative ligand that activates the β-catenin independent pathway in Wnt signaling, is involved in the pathogenesis of osteoarthritis (OA)[65, 66, 68].

Using immunohistological analysis, Li et al. reported that Wnt-5a was expressed in human NP tissues and that its expression was significantly elevated in degenerated human NP tissues[65]. Interestingly, recent studies showed that Wnt/β-catenin signaling pathway was activated by YAP1, a downstream nuclear effector of the Hippo signaling pathway[69, 70], which was also identified to be differentially methylated in the current study.

The results of the current study suggest the possibility that genes for catabolic molecules, including pro-inflammatory cytokines and matrix-degrading enzymes, may not be differentially methylated during disc degeneration in humans. However, DNA methylation may be differentially regulated in genes associated with signaling pathways, such as NF-κB, MAPK-ERK and Wnt signaling pathways, that are located upstream to the gene transcription of these catabolic molecules.

It is well known that the anabolic regulators of human IVD cells include polypeptide growth factors, such as IGF-1, transforming growth factor-β (TGF-β), and the bone morphogenetic proteins (BMPs)[71, 72].

SMADS, the main signal transducers for receptors of TGF-β[73], play important roles in stimulating cell proliferation and IVD cell matrix metabolism. We found that *SMAD3* was differentially methylated in the IVD degeneration stage compared to that at the early stage. A previous study showed that TGF-β upregulated aggrecan and sulfated glycosaminoglycans (sGAG) synthesis [64]. sGAG synthesis was recently reported to be stimulated by the SAMD3 signaling pathway in the regulation of expression of β-1,3-glucuronosyltransferase 1 (GlcAT-1), a key enzyme that catalyzes glycosaminoglycan (GAG) synthesis[74, 75]. This suggests that SMAD3 may be implicated in IVD degeneration. Additionally, a differential methylation level of other genes that regulate TGF-β signaling, such as *MECOM* and *ELAC2*, was also identified in our study.

Interestingly, other important growth factor-related genes, such as *IGFBP4* and *FGFBP2*, were found to be differentially methylated in the advanced IVD degeneration stage compared with the early stage. From these results, we can speculate that DNA methylation profiles may be differentially regulated, not only in catabolic factors, but also in anabolic factors, such as growth factors, that can regulate cell proliferation and extracellular matrix metabolism of the human NP.

Our results also showed that several enzymes that catalyze the biosynthesis of sulfated glycosaminoglycans (GAGs) of proteoglycans, including *CHST1*, *EXTL3* and *SLC26A2*, were differentially methylated in the advanced stage of human disc degeneration compared to those in the early stage.

In our study, three genes associated with the regulation of Hedgehog (Hh) signaling were also found to be differentially methylated in the advanced IVD degeneration stage, including *SUFU*[76–78], *TTCIB[79]* and *IQCTH[80]*. Hh signaling plays pivotal roles in regulating normal chondrocyte growth and differentiation. Lin et al. recently reported that a higher level of Hh signaling in chondrocytes is responsible for the severe osteoarthritis phenotype, suggesting that Hh signaling is associated with the severity of OA[81]. Furthermore, Sonic hedgehog (Shh), secreted by NP cells, is essential for cell proliferation in the growing disc and differentiation in the developmental stage of the mouse IVD[82]. Therefore, our results also suggest that changes in methylation profiles related to the hedgehog pathway may be responsible for the development of disc degeneration.

The GO enrichment analysis of differentially methylated genes further revealed significant GO terms for biological processes associated with cell adhesion; hemophilic cell adhesion through plasma membrane adhesion molecules and cell-cell adhesion through plasma membrane adhesion molecules. Cell-matrix interactions of NP cells, as well as chondrocytes, play crucial roles in regulating several functions, including cell survival and matrix metabolism, acting through anabolic and catabolic signaling pathways through integrin and other ECM receptors[83–86]. The results of the current study suggest that differential methylation loci may not accumulate in ECM molecules and/or catabolic molecules themselves, but would rather accumulate in the molecules associated with cell-matrix and/or cell-cell adhesion that are related to the major signaling pathways relevant to the process of human disc degeneration.

Human IVDs and articular cartilage share remarkably similar anatomical composition, biochemical features and molecular processes of matrix degeneration. Genetically, these two matrix degenerative states also have common susceptibility alleles, such as single-nucleotide polymorphism rs143383 in the 5’ untranslated region of *GDF5* and asporin D14 triplet repeat[87]. Therefore, we compared the DNA methylation profiles of human IVD degeneration (data from the current study) with those from a previous study of human knee OA[44]. The results of this analysis showed that 2.7% (6/220) of DMLs overlapped between these two diseases. The overlapping genes include *YAP1*[69, 70] and *FGFRL1*[88–91], which have been reported to be associated with the pathogenesis of disc degeneration and OA. Although these two diseases share common pathological features, the DNA methylation profiles were very different. The authors speculate that, in addition to the genetic background, anatomical differences in the mobile joint structure of IVDs and knee joints would contribute to differences in DNA methylation profiles between these two diseases during the process of tissue degeneration.

There were some limitations to this study. First, IVD samples with early stage disc degeneration were difficult to obtain from spine surgeries. Most samples of MRI grades I to III were obtained from spinal trauma surgeries of relatively young patients. Because of the small number of samples, IVDs with Pfirrmann MRI grades I to III were all grouped as early stage degeneration. Differences in DNA methylation patterns among these three grades, which would be associated with the initiation of human disc degeneration, should be evaluated in a future study. Second, because the radiological, biochemical and histological features of degenerative changes in human NP tissues were well characterized[9], human NP tissues were isolated and processed for DNA methylation analysis in this study. On the other hand, AF tissues are also known to show degenerative changes, including irregularity of the lamella and collagen degeneration[9]. Therefore, it would also be of great importance to evaluate the DNA methylation profile of human AF tissues between early and advanced stages of disc degeneration in a future study. Third, recent epigenetic studies have shown that age and gender are significantly associated with the changes in DNA methylation profiles [92–95]. Since differences in age and gender inequality exist between ED and AD group, there would be the possibility that these two factors may have potential to affect the DNA methylation profiles of human IVD degeneration in this study. Forth limitation of this study is that the expression of individual hypomethylated and hypermethylated genes was not examined in this study. It has been reported that gene transcription is also influenced by the gene features (CpG-dense promotors or gene body) where methylation occurs[13]. Therefore, the genome-wide gene expression analysis, such as RNA sequencing would be needed for further evaluating the function of DNA methylation in the process of human IVD degeneration in a future study.

## Conclusion

We conducted, for the first time, a genome-wide DNA methylation profile comparative study and observed significant differences in DNA methylation profiles between early and advanced stages of human IVD degeneration.

The overview of the DNA methylation profile in the current study revealed that DMLs were identified in many genes associated with known molecules that have been reported to be relevant to IVD degeneration. Importantly, changes in DNA methylation profiles were also found in genes that regulate the major signaling pathways, such as NF-κB, MAPK, and Wnt signaling, that are well known to be responsible for the pathogenesis of human disc degeneration.

According to the GO analysis, DMLs tended to accumulate in molecules associated with cell adhesion, suggesting that diverse signaling pathways that regulate the cell-ECM or cell-cell interactions that orchestrate cell survival and matrix metabolism may be implicated in the process of human IVD degeneration.

Since the results of this study are still preliminary in a small number of samples, the evaluation of gene and protein expression in addition to a genome-wide DNA methylation profiles in an increasing number of samples would be needed to elucidate the pathological mechanism of human IVD degeneration in a future study.

## Supporting information

S1 Table Complete list of significantly differentially methylated loci (DMLs). (XLSX)

S1 Fig Differences in the β value of the four highest hypomethylated and hypermethylated loci among each grade of degeneration (Pfirrmann grades I-IV[32]). One- way ANOVA was used to compare the β value of each grade’s samples. Pairwise comparisons were conducted with Bonferroni post hoc correction. * = P < 0.05; ** = P < 0.01; *** = P < 0.001. A: CARD14 (Caspase Recruitment Domain Family Member 14), B: CRHR1 (Corticotropin Releasing Hormone Receptor 1), C: C14orf139 (Chromosome 14 Open Reading Frame 139), D: ZBTB47 (Zinc Finger And BTB Domain Containing 47), E: GNL3 (G Protein Nucleolar 3), F: SNORA52 (Small Nucleolar RNA, H/ACA Box 52), G: XKR5 (X Kell Blood Group Precursor-Related Family, Member 5), H: MED23 (Mediator Complex Subunit 23).
(TIFF)
