# Supplementary figures and images for: Genome-wide analysis of DNA methylation profile identifies differentially methylated loci associated with human intervertebral disc degeneration
Source: PLoS One. 2019 Sep 12;14(9):e0222188. doi: 10.1371/journal.pone.0222188 (PMC6742346; doi:10.1371/journal.pone.0222188)

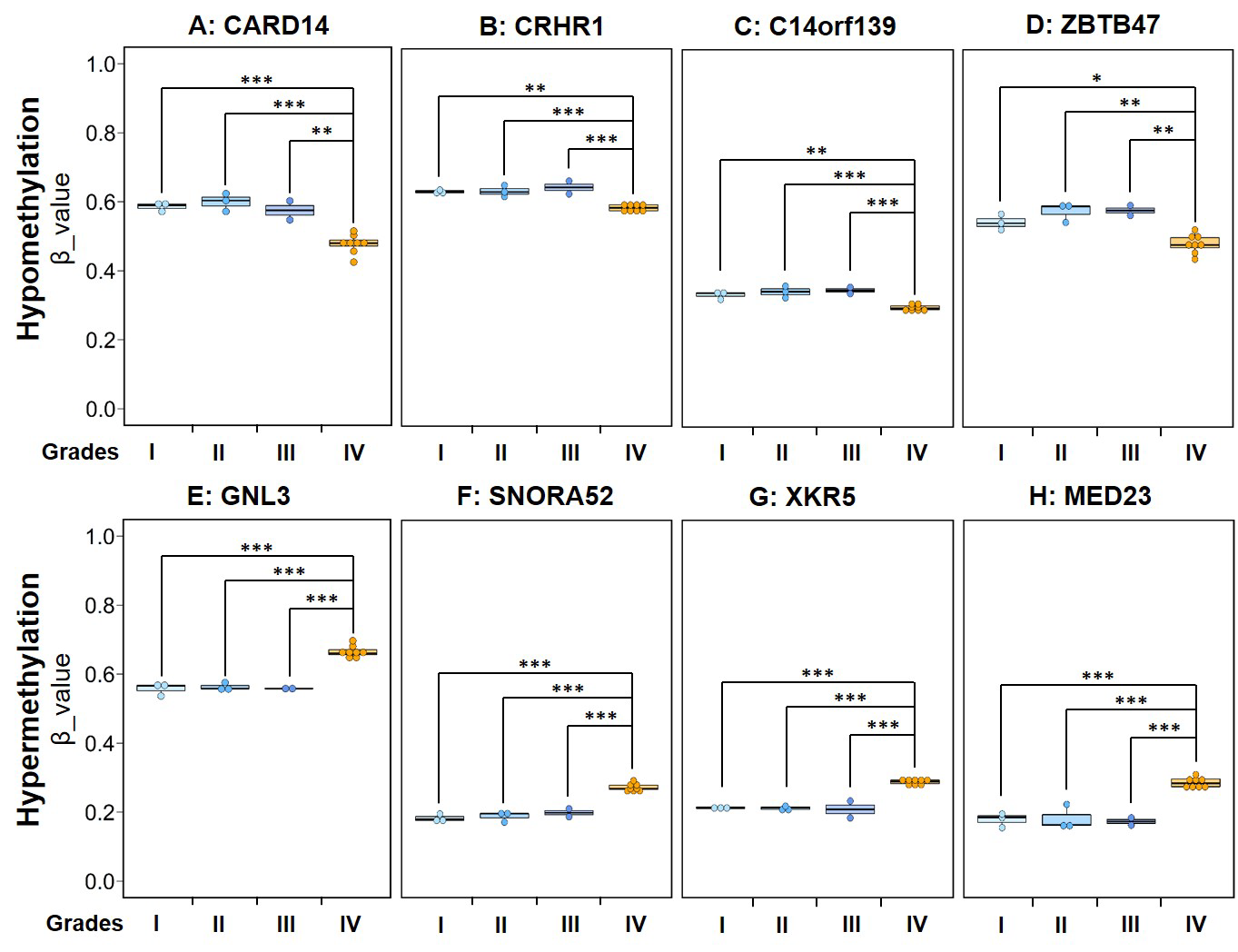

Supplement: S1 Fig — One- way ANOVA was used to compare the β value of each grade’s samples. Pairwise comparisons were conducted with Bonferroni post hoc correction. * = P < 0.05; ** = P < 0.01; *** = P < 0.001. A: CARD14 (Caspase Recruitment Domain Family Member 14), B: CRHR1 (Corticotropin Releasing Hormone Receptor 1), C: C14orf139 (Chromosome 14 Open Reading Frame 139), D: ZBTB47 (Zinc Finger And BTB Domain Containing 47), E: GNL3 (G Protein Nucleolar 3), F: SNORA52 (Small Nucleolar RNA, H/ACA Box 52), G: XKR5 (X Kell Blood Group Precursor-Related Family, Member 5), H: MED23 (Mediator Complex Subunit 23). (TIFF) [file pone.0222188.s002.tiff]
